# Supplementary material for: Anomalous frozen evanescent phonons
Source: Nat Commun. 2024 Oct 24;15:8882. doi: 10.1038/s41467-024-52956-5 (PMC11502830; doi:10.1038/s41467-024-52956-5)
Supplement: Supplementary file 1 — Supplementary Information [file 41467_2024_52956_MOESM1_ESM.pdf]

Anomalous frozen evanescent phonons – Supplementary Information

Yi Chen[1, 2]†*, Jonathan L.G. Schneider[2]†, Ke Wang[2,3]†, Philip Scott[2], Sebastian Kalt[2], Muamer Kadic[4], and Martin Wegener[1,2]*

†These authors have contributed equally.

[1]Institute of Nanotechnology, Karlsruhe Institute of Technology (KIT), Karlsruhe 76128, Germany.

[2]Institute of Applied Physics, Karlsruhe Institute of Technology (KIT), Karlsruhe 76128, Germany.

[3]National Key Laboratory of Science and Technology on Advanced Composites in Special Environments, Harbin Institute of Technology, Harbin 150001, China.

[4]Université de Franche-Comté, Institut FEMTO-ST, UMR 6174, CNRS, Besançon 25000, France.

[*]Corresponding authors: yi.chen@partner.kit.edu (Y.C.); martin.wegener@kit.edu (M.W.)

Supplementary Figures and Captions

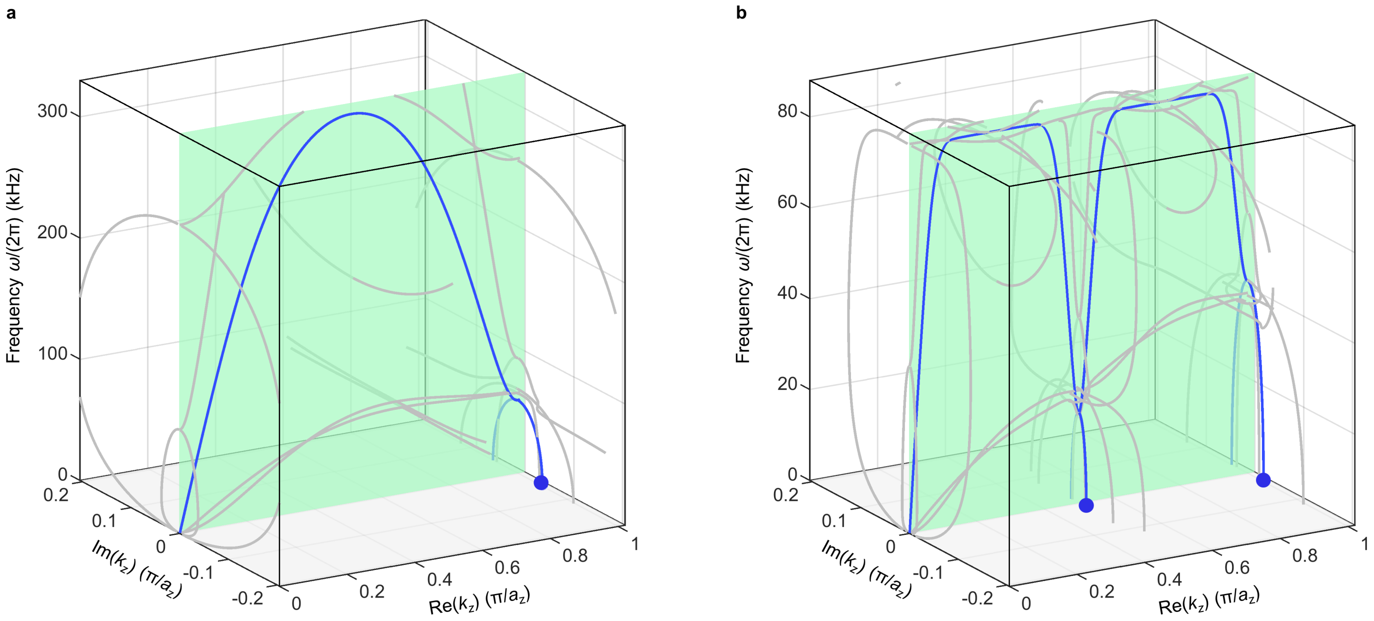

Supplementary Figure 1. Same as Fig. 3a but for metamaterials with (a) $N = 2$ and (b) $N = 4$. For $N = 2$, the longitudinal branch (blue curve in (a)) exhibits a local minimum at the edge of the first Brillouin zone, leading to a frozen evanescent mode with wavenumber $k_z = (1 - 0.044\ \mathrm{i})\pi/a_z$ (cf. blue dot in (a)). In contrast, the longitudinal branch for $N = 4$ (blue curve in (b)) shows two local minima and two resulting frozen evanescent modes at wavenumbers, $k_z = (0.5 - 0.013\ \mathrm{i})\pi/a_z$ and $k_z = (1 - 0.033\ \mathrm{i})\pi/a_z$ (cf. two blue dots in (b)).

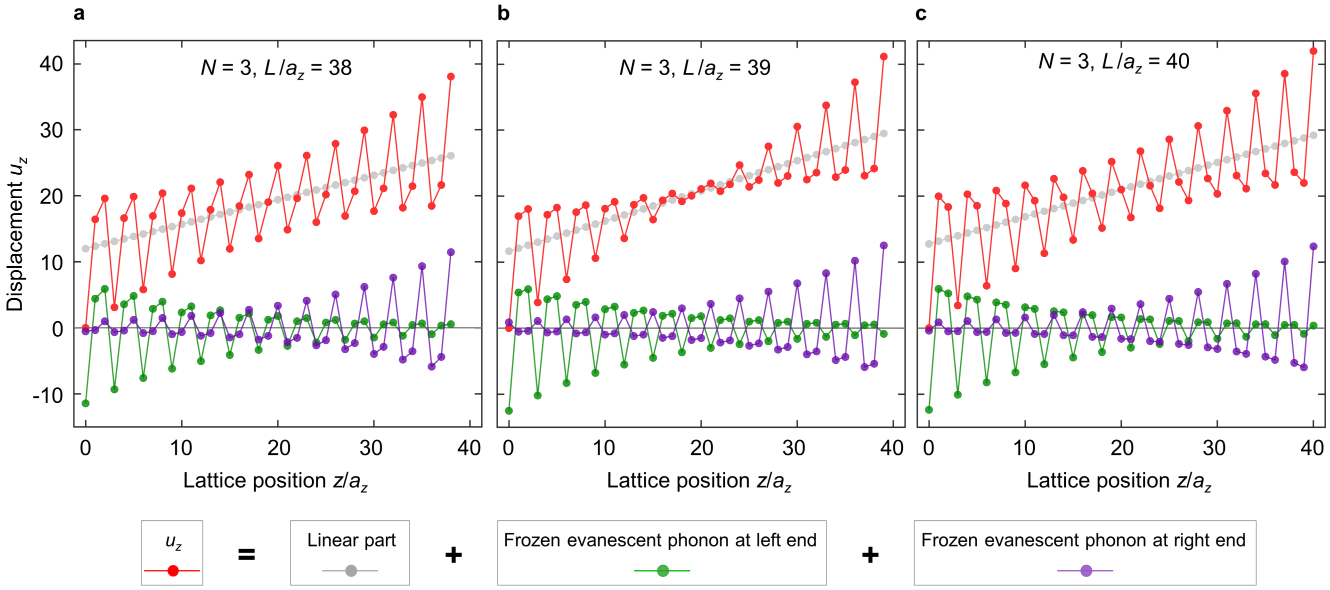

Supplementary Figure 2. Same as Fig. 6a but for (a) $N = 3, L/a_z = 38$, (b) $N = 3, L/a_z = 39$, and (c) $N = 3, L/a_z = 40$.

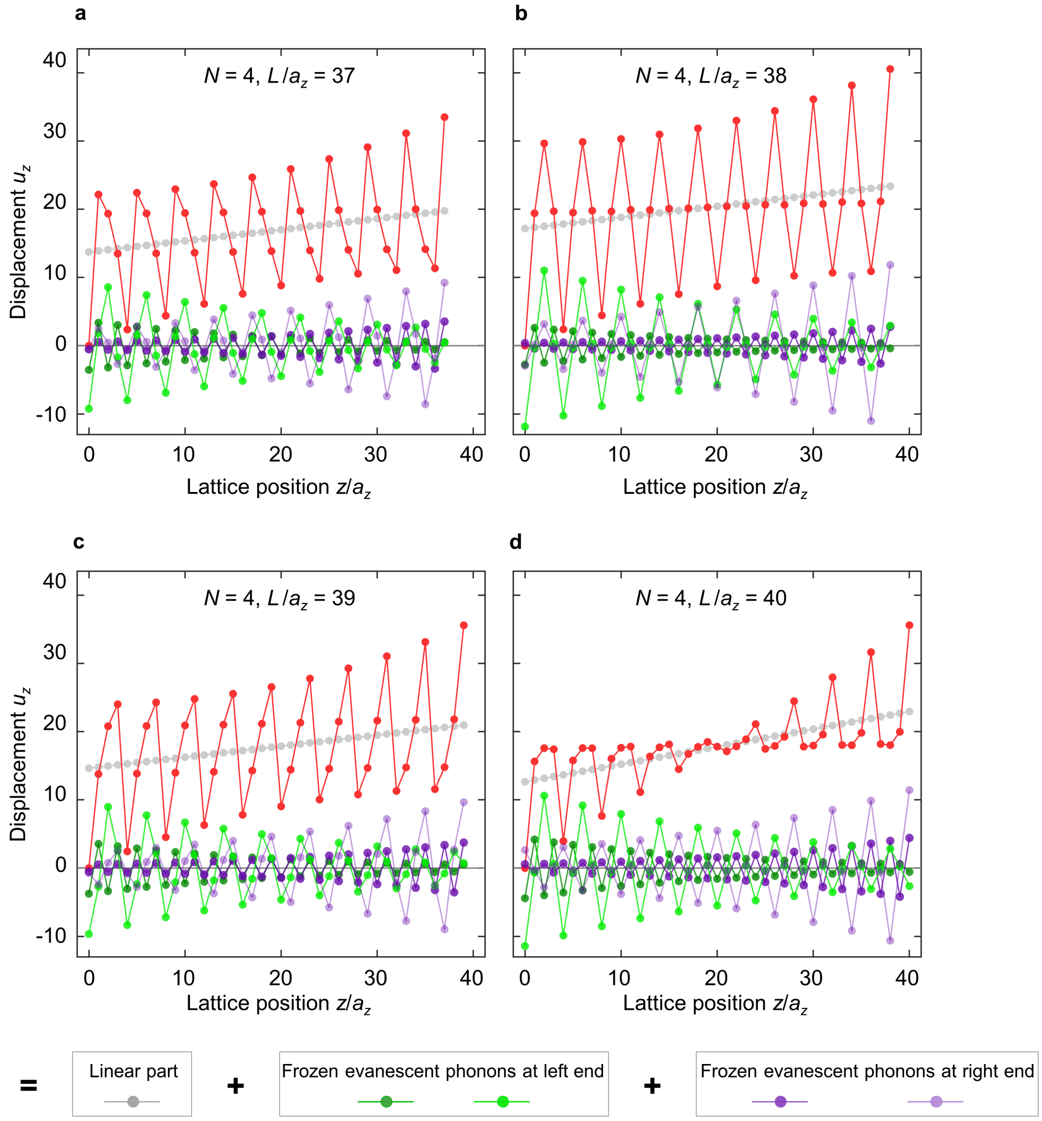

Supplementary Figure 3. Same as Fig. 6a, but for (a) $N = 4, L/a_z = 37$, (b) $N = 4, L/a_z = 38$, (c) $N = 4, L/a_z = 39$, and (d) $N = 4, L/a_z = 40$. We note that there are two instead of one frozen evanescent phonon eigensolutions localized at the two ends of the finite-length beam. One has an oscillation period of $2a_z$ (cf. green and purple dots), while the other oscillates with a period of $4a_z$ (cf. light green and light purple dots), consistent with the complex wavenumbers for the frozen evanescent phonons (cf. Methods in the main paper).

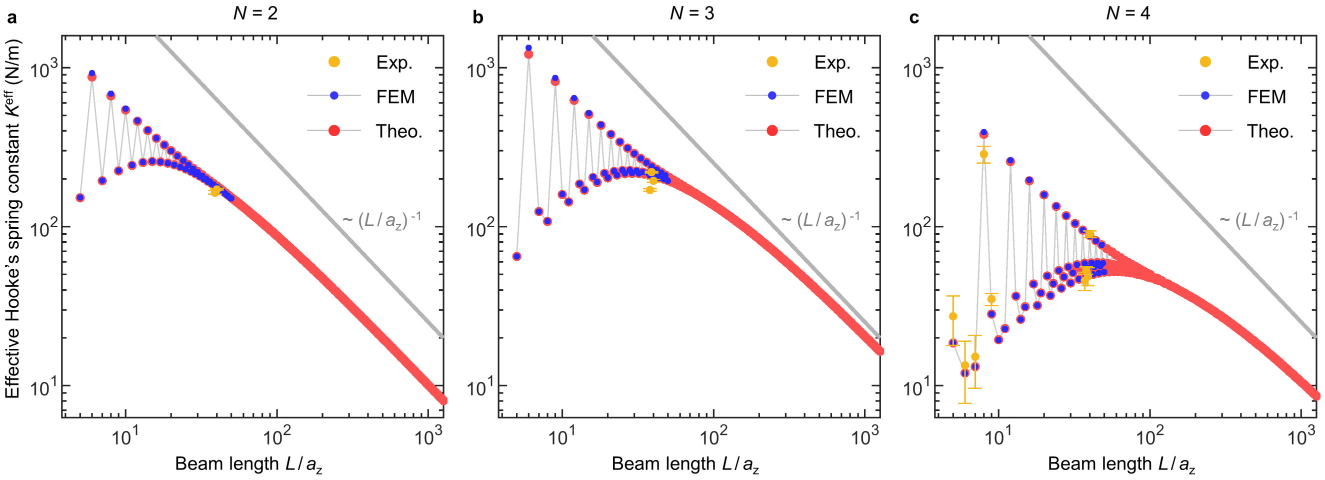

Supplementary Figure 4. Non-monotonic effective Hooke's spring constant versus metamaterial-beam length. (a) $N = 2$. The effective spring constant is calculated via $K^{\text{eff}} = F_z/u_{\text{max}}$, with u_{max} being the displacement at the metamaterial beam end and F_z being the z-component of the loading force vector. Blue (red) dots represent finite-element calculations (mass-and-spring model). Measured stiffness constants corresponding to the samples in Fig. 5 are shown as yellow dots together with statistical error bars (standard deviation) of the measurements. The thin gray lines in between the dots are added for clarity. The effective spring constant versus L/a_z exhibits an oscillation with period $N = 2$. If the normalized beam length L/a_z is commensurable with $N = 2$, the effective stiffness roughly scales as $\propto 1/L$ (cf. thick gray line), following an ordinary (Cauchy) elastic material. Otherwise, the stiffness increases before reaching a maximum at a characteristic length $\approx 2l/a_z$, and then decreases $\propto 1/L$. This anomalous behavior originates from the interference of four different frozen evanescent phonons in the metamaterial beams. (b) and (c) Same as (a), but for $N = 3$ and $N = 4$, respectively. For $N = 4$, we have fabricated and characterized additional metamaterial beams with lengths $L/a_z = 5, 6, 7,\ 8$ and presented the experimentally measured effective spring constants. The overall agreement between experiment and theory is good.

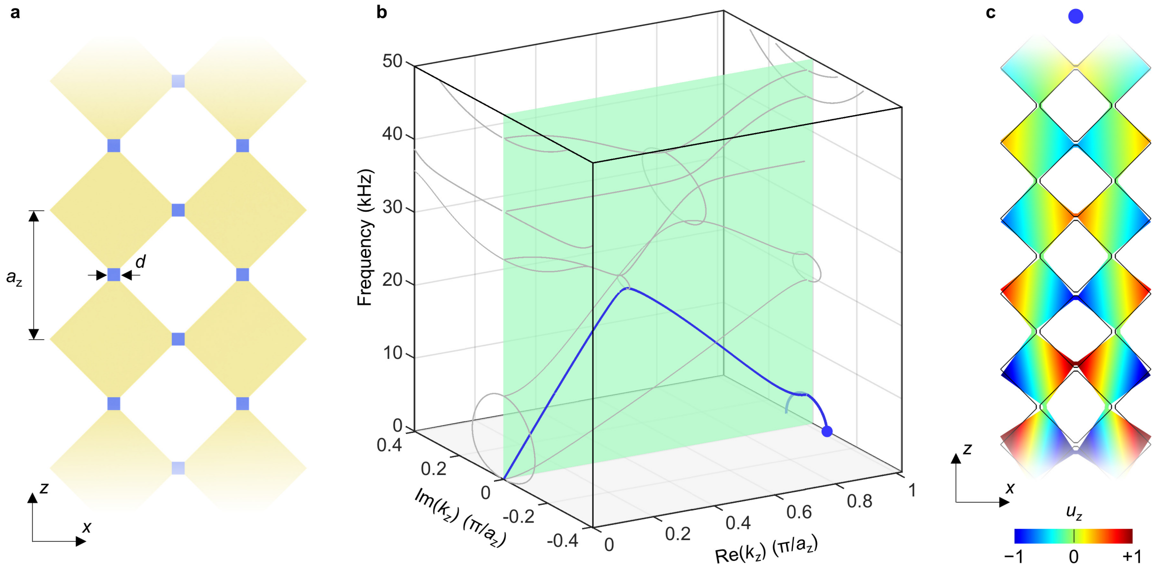

Supplementary Figure 5. Another example of anomalous frozen evanescent modes in mechanism-based mechanical metamaterial. (a) Metamaterial [1] consisting of squares (light yellow) connected by non-ideal hinges (cf. small blue squares). The colors are for illustration only; all is made from a single constituent material. The hinges become ideal if the blue squares become infinitely small, leading to a floppy mode (with $\mathrm{Im}(k_z) = 0$ at $\mathrm{Re}(k_z) = \pi/a_z$) or mechanism of deformation (neighboring squares rotate oppositely) at zero energy cost. (b) Calculated complex-valued phonon band structures of the metamaterial. Here, we choose the same constituent-material parameters as in Fig. 3 and the geometry parameters $d/a_z = 0.05$ and $a_z = 1$ cm. The wavevector, $\mathbf{k} = (0,0,k_z)$, is along the beam axis. Frozen evanescent phonon modes ($k_z = (1 + 0.09\,\mathrm{i})\,\pi/a_z$ (cf. blue dot in (b)) emerge from the low-frequency local minimum of the blue band at the edge of the first Brillouin zone. The local minimum further approaches zero frequency for yet smaller ratios d/a_z (not depicted). (c) Displacement field of the frozen evanescent mode (cf. blue dot in (b)). Neighboring squares along the z-direction rotate in opposite directions, consistent with the period of $p = 2\pi/\mathrm{Re}(k_z) = 2a_z$.

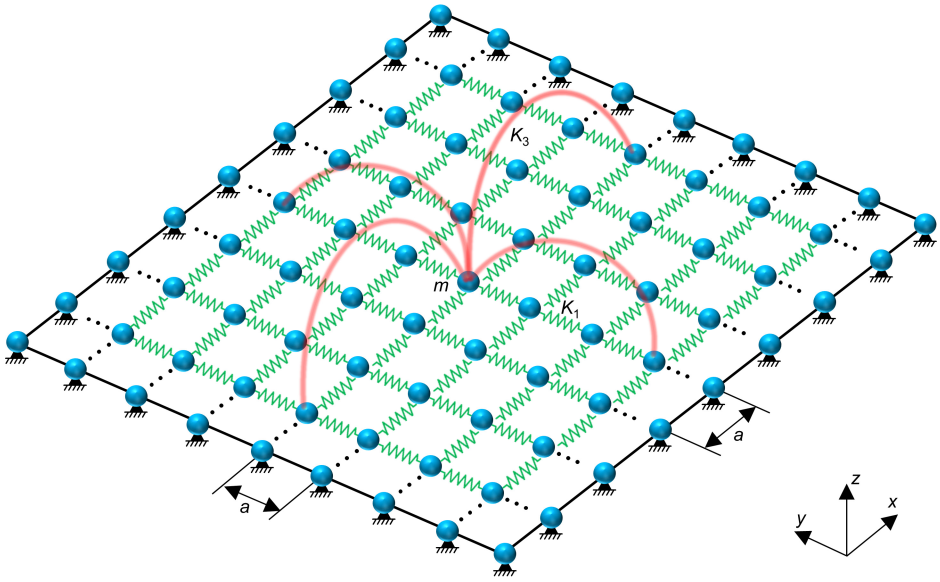

Supplementary Figure 6. Two-dimensional (2D) nonlocal periodic mass-and-spring model. Each mass m (blue dots) is connected to its four nearest neighbors along the x- and y-direction via Hooke's springs (cf. green zigzag curves), with spring constant K_1. Furthermore, we couple each mass to its third-nearest-neighbors by nonlocal springs (for the example of $N = 3$), symbolized by the red curves, with spring constant K_3. For clarity, only four selected third-nearest-neighbor couplings are shown. We consider out-of-plane displacements, u_z, of the masses. The masses at the four edges of the model are fixed, mimicking a suspended elastic membrane of a drum.

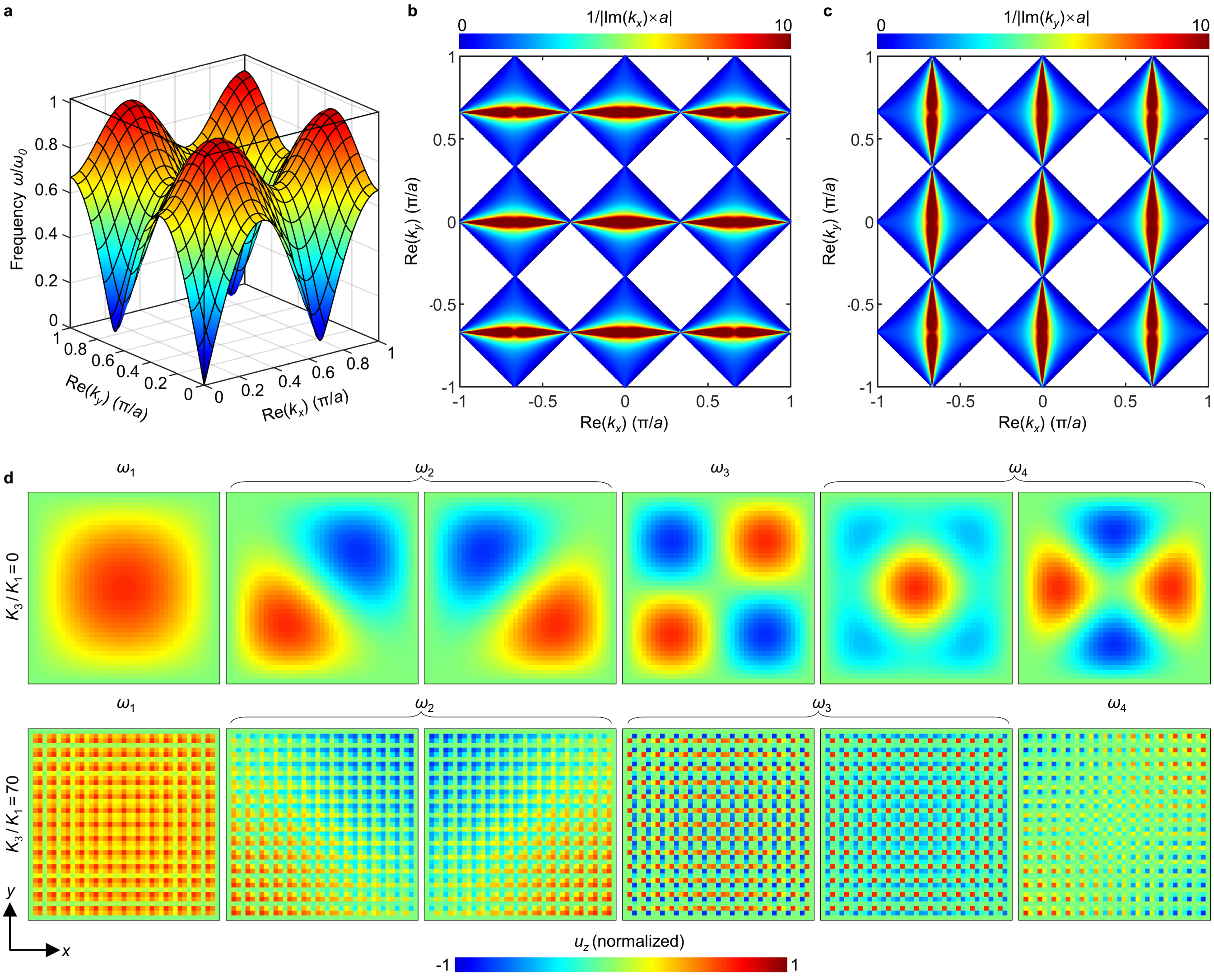

Supplementary Figure 7. Illustration of frozen evanescent Bloch modes in a 2D nonlocal system. (a) Calculated normalized eigenfrequency $\omega(k_x, k_y)/\omega_0$ versus the wavenumbers k_x and k_y for the 2D model illustrated in Supplementary Fig. 6. The parameters chosen are: $N = 3$, $K_3/K_1 = 70$, and $\omega_0 = \sqrt{(K_1 + 9K_3)/m}$. The part with $\mathrm{Re}(k_x) < 0$ and $\mathrm{Re}(k_y) < 0$ can be obtained by mirror reflecting the plot. The strong nonlocal interactions lead to multiple local minima of the frequency surface. (b) Solutions of wavenumbers corresponding to frozen evanescent Bloch modes. White regions represent the absence of solutions for frozen evanescent Bloch modes. Color represents the normalized decaying length $l/a = 1/|\mathrm{Im}(k_x)a|$. In sharp contrast to isolated frozen evanescent modes in 1D periodic system (cf. Fig. 3a), the wavenumbers of frozen evanescent modes in the 2D system form compact regions in the space of $\mathrm{Re}(k_x)$ and $\mathrm{Re}(k_y)$. Each region is centered at the wavenumber $(\mathrm{Re}(k_x),\ \mathrm{Re}(k_y))$, which corresponds to a local minimum of the eigenfrequency $\omega(\mathrm{Re}(k_x),\ \mathrm{Re}(k_y))$ (cf. panel (a)). (c) Same as (b) but the false-color represents $l/a = 1/|\mathrm{Im}(k_y)a|$. (d) First six eigenmodes of a finite 2D model (side length $40a$) with four edges fixed (cf. Supplementary Fig. 4). The first row corresponds to a local system ($K_3/K_1 = 0$) and the second row to a nonlocal system ($K_3/K_1 = 70$). All eigenmodes for the nonlocal model, including the fundamental (first) eigenmode, exhibit pronounced oscillations with a spatial period of $3a$.

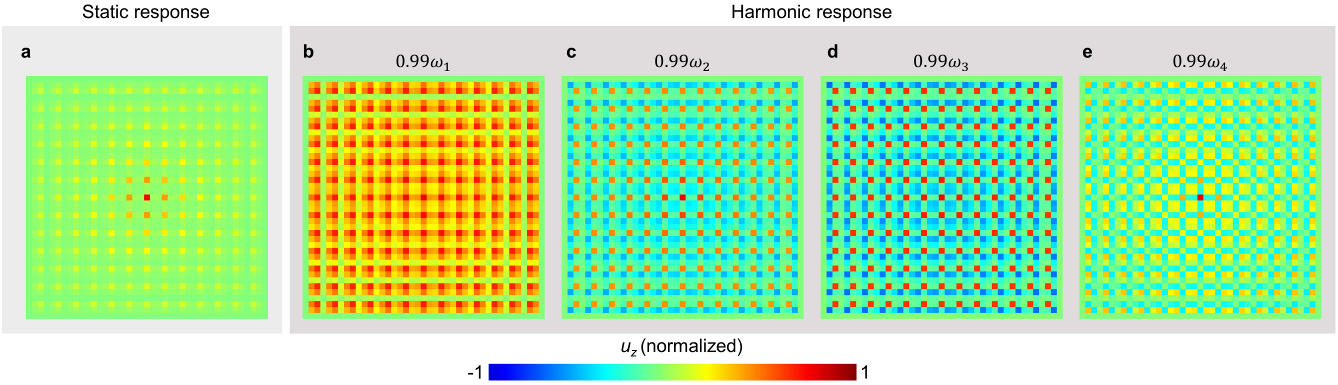

Supplementary Figure 8. Static and dynamic response of the 2D nonlocal system in Supplementary Fig. 6. (a) Displacement field of the system under static loading at the center of the membrane. The four edges of the system are fixed. (b)-(e) Same as (a), but for harmonic time-harmonic excitation at a frequency close to one of the first four eigenfrequencies, i.e., $0.99\omega_1$, $0.99\omega_2$, $0.99\omega_3$, $0.99\omega_4$, respectively (in the absence of damping, exciting the system exactly at an eigenfrequency leads to a divergence). In both static case and dynamic case, spatial oscillations with period $3a$ are observed – as for the eigenmodes shown in Supplementary Fig. 7d.

Supplementary References

[1] C. Coulais, C. Kettenis, and M. van Hecke, A characteristic lengthscale causes anomalous size effects and boundary programmability in mechanical metamaterials. Nat. Phys. 14, 40 (2017).
