# Supplementary material for: Anomalous frozen evanescent phonons
Source: Nat Commun. 2024 Oct 24;15:8882. doi: 10.1038/s41467-024-52956-5 (PMC11502830; doi:10.1038/s41467-024-52956-5)
Supplement: Supplementary file 3 — Description of Additional Supplementary Files [file 41467_2024_52956_MOESM3_ESM.pdf]

Description of Additional Supplementary Files

Supplementary Movie 1: One loading-unloading cycle of the stretching experiment performed on a metamaterial beam with $N = 2$.

Supplementary Movie 2: Zoom-in view at the top of the metamaterial in Supplementary Movie 1. A spatial oscillation period $2a_z$ for the displacement can be seen due to the excited frozen evanescent phonons. It is also clearly seen that some local springs are compressed despite the sample is stretched.

Supplementary Movie 3: One loading-unloading cycle of the stretching experiment performed on a metamaterial beam with $N = 3$.

Supplementary Movie 4: Zoom-in view at the top of the metamaterial in Supplementary Movie 3. A spatial oscillation period $3a_z$ for the displacement can be seen due to the excited frozen evanescent phonons. It is also clearly seen that some local springs are compressed despite the sample is stretched.

Supplementary Movie 5: One loading-unloading cycle of the stretching experiment performed on a metamaterial beam with $N = 4$.

Supplementary Movie 6: Zoom-in view at the top of the metamaterial in Supplementary Movie 5. A spatial oscillation period $4a_z$ for the displacement can be seen due to the excited frozen evanescent phonons. It is also clearly seen that some local springs are compressed despite the sample is stretched.

Supplementary Movie 7: One loading-unloading cycle of the stretching experiment performed on a metamaterial beam with $N = 2$. The last two sites are loaded simultaneously as in Fig. 5d.

Supplementary Movie 8: Zoom-in view at the top of the metamaterial in Supplementary Movie 7. The displacement doesn't exhibit oscillation period of $2a_z$ since the frozen evanescent phonons at the top are suppressed by the two-sites loading.

Supplementary Movie 9: Zoom-in view at the bottom of the metamaterial in Supplementary Movie 7. In contrast to the displacement at the top of the metamaterial beam, an oscillation period of $2a_z$ can be observed at the bottom.

Supplementary Movie 10: Another view of the stretching experiment for a metamaterial with $N = 3$. The hook can clearly be seen.
